# Supplementary material for: Effectiveness of Wolbachia-infected mosquito deployments in reducing the incidence of dengue and other Aedes-borne diseases in Niterói, Brazil: A quasi-experimental study
Source: PLoS Negl Trop Dis. 2021 Jul 12;15(7):e0009556. doi: 10.1371/journal.pntd.0009556 (PMC8297942; doi:10.1371/journal.pntd.0009556)
Supplement: S1 Text — (DOCX) [file pntd.0009556.s001.docx]

**kdr genotyping**

Adult *Aedes aegypti* were genotyped by qPCR to detect single nucleotide polymorphisms (SNPs) at the 1016 (Val+ or Ilekdr) and 1534 (Phe+ or Cyskdr) positions of the voltage gated sodium channel gene (NaV), as previously reported [1,2]. Amplification reaction was performed with LightCycler 480 Probes Master mix (Roche), 10ng of individual genomic DNA, and a set of primers and probes to detect kdr alleles (below) customized by Thermo Fisher Inc. under ID codes: AHS1DL6 (Val+1016Ilekdr) and AHUADFA (Phe+1534Cys). Thermal cycling was carried out on a Light Cycler 480 Instrument II (Roche), set to the following conditions: 95 ºC for 10 min (initial denaturation), and N cycles of 95 ºC for 15 s and 60 ºC for 30 s (single acquisition). N was set to 30, for amplifying Val+1016Ilekdr, or to 40, for Phe+1534Cyskdr. For each collection date, up to 90 samples were individually genotyped.

**References**

1. Hayd RLN, Carrara L, de Melo Lima J, de Almeida NCV, Lima JBP, Martins AJ. Evaluation of resistance to pyrethroid and organophosphate adulticides and kdr genotyping in *Aedes aegypti* populations from Roraima, the northernmost Brazilian State. Parasit Vectors 2020;13:264.

2. Macoris ML, Martins AJ, Andrighetti MTM, Lima JBP, Valle D. Pyrethroid resistance persists after ten years without usage against *Aedes aegypti* in governmental campaigns: Lessons from Sao Paulo State, Brazil. PLoS Negl Trop Dis 2018;12:e0006390.

**Nucleotide sequences of primers and probes.** List of primers and probes used for the molecular diagnostics of *Wolbachia* by qPCR and LAMP, and for kdr genotyping.

| PRIMER | NUCLEOTIDE SEQUENCE (5’ à 3’) |
| --- | --- |
| Ae. aegypti RPS17 – qPCR | |
| RPS17S Forward | TCCGTGGTATCTCCATCAAGCT |
| RPS17S Reverse | CACTTCCGGCACGTAGTTGTC |
| RPS17S Probe | HEX/CAGGAGGAG/ZEN/GAACGTGAGCGCAG/3lABkFQ |
| Ae. aegypti kdr screening – qPCR | |
| 1016 Forward | CGTGCTAACCGACAAATTGTTTCC |
| 1016 Reverse | GACAAAAGCAAGGCTAAGAAAAGGT |
| 1016 Probe Val+ | VIC/CCGCACAGATACTTA/NFQ |
| 1016 Probe Ilekdr | FAM/CCCGCACAGGTACTTA/NFQ |
| 1534 Forward | CGAGACCAACATCTACATGTACCT |
| 1534 Reverse | GATGATGACACCGATGAACAGATTC |
| 1534 Probe Phe+ | FAM/ACGACCCGAAGATGA/NFQ |
| 1534 probe Cyskdr | VIC/AACGACCCGCAGATGA/NFQ |
| Wolbachia – qPCR | |
| WSPTM2 Forward | CATTGGTGTTGGTGTTGGTG |
| WSPTM2 Reverse | ACACCAGCTTTTACTTGACCAG |
| WSPTM2 Probe | FAM/TCCTTTGGA/ZEN/ACCCGCTGTGAATGA/3lAbRQSp |
| Wolbachia – LAMP | |
| FIP | TGTATGCGCCTGCATCAGCTTCGGTTCTTATGGTGCTAA |
| BIP | GCAGAAGCTGGAGTAGCGTTGTGTCATGCCACTTAGATGG |
| F3 | TGATGTAACTCCAGAAGTCA |
| B3 | CTTATTGGACCAACAGGATCG |
| LpF | AGCCTGTCCGGTTGAATT |
| LpB | CAGTCTTGTTATCCCAGTGAGT |
