# Supplementary figures and images for: Effectiveness of Wolbachia-infected mosquito deployments in reducing the incidence of dengue and other Aedes-borne diseases in Niterói, Brazil: A quasi-experimental study
Source: PLoS Negl Trop Dis. 2021 Jul 12;15(7):e0009556. doi: 10.1371/journal.pntd.0009556 (PMC8297942; doi:10.1371/journal.pntd.0009556)

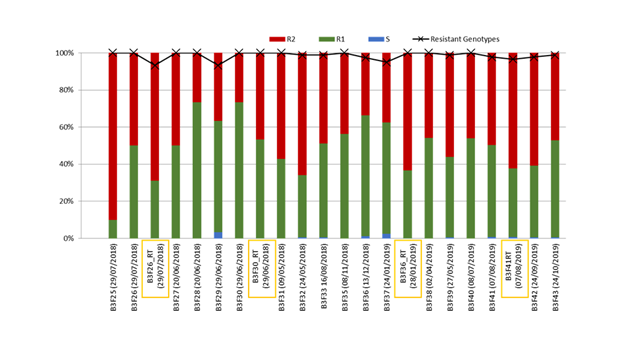

Supplement: S1 Fig — Outcrossing events were performed on brood females in generation F26, F30, F36 and F41. ‘_RT’ represents the offspring of those outcrossing events and are marked with an orange box. Field collected samples in Rio and Niteroi always show highly resistant genotypes with a roughly 50:50 frequency distribution of R1 and R2 mutations. The wMelRio brood stock line tends to increase its R1 frequency with standard inbred rearing and some small % of susceptible genotypes start to appear around the 3rd Generation. The resulting outcross event normally restores the near 50:50 R1:R2 frequency distribution and reduces susceptible genotype frequencies as well. Methods for kdr genotyping, primers and probes are presented in supplementary methods. (TIF) [file pntd.0009556.s002.tif]

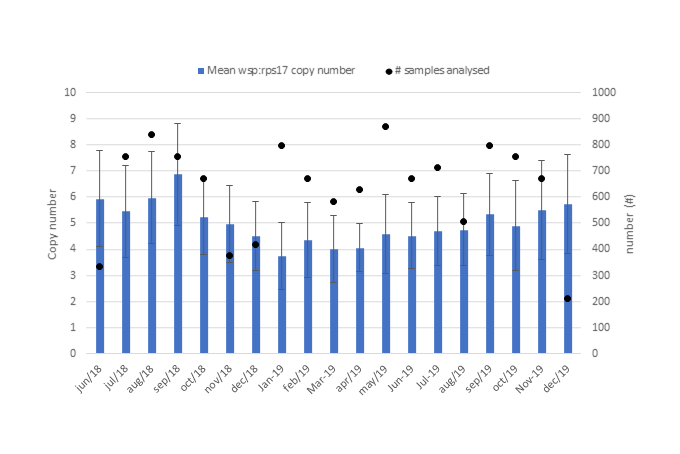

Supplement: S2 Fig — Quantification of wMel was performed weekly on up to 4 day old mosquitoes from the release generation, emerged within the release device, prior to releases. wsp:rps17 copy numbers were fairly constant between 4 to 6, from June 2018 to December 2019. Error bars represent standard deviation of the mean. Total numbers of mosquitoes tested are represented by black dots. (TIF) [file pntd.0009556.s003.tif]

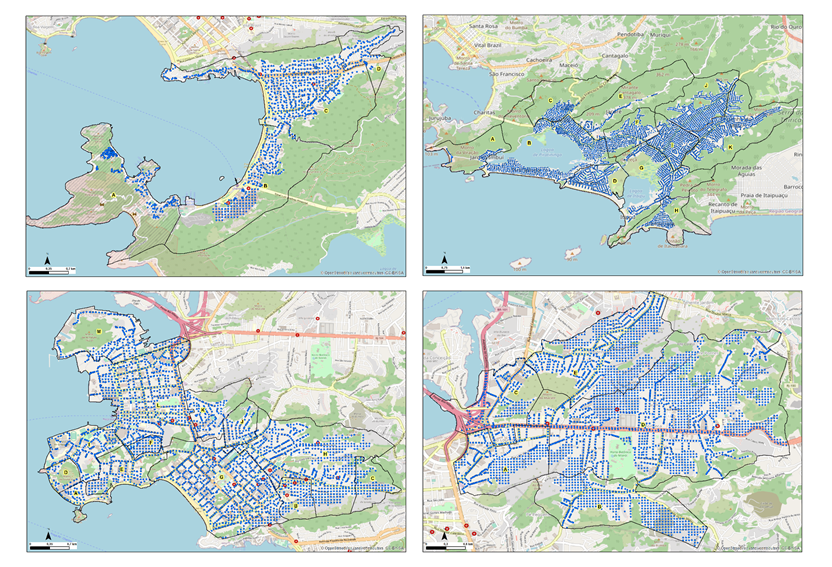

Supplement: S3 Fig — Spatial distribution of mosquito release locations in Niterói release zone 1 (A), zone 2 (B), zone 3 (C) and zone 4 (D). Approximate locations of adult mosquito releases are shown by blue markers. The Jurujuba pilot release area in zone 1 is indicated with hatched shading. Maps were generated in ArcGIS 10.7 (Esri, Redlands, CA, USA) using base map and data from OpenStreetMap under open database license (CC BY-SA). (TIF) [file pntd.0009556.s004.tif]

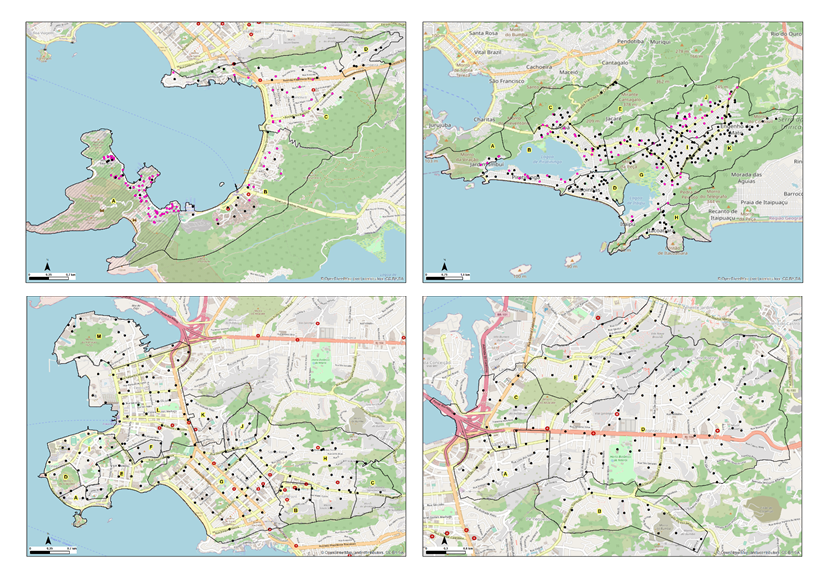

Supplement: S4 Fig — Spatial distribution of mosquito monitoring locations in Niterói release zone 1 (A), zone 2 (B), zone 3 (C) and zone 4 (D). Approximate locations of BG adult mosquito traps are shown for each zone. Black markers indicate BG traps that were retained throughout the monitoring period. Pink markers indicate BG traps that were removed in three of four neighbourhoods in zone 1 and six of 11 neighbourhoods in zone 2 once releases were completed and wMel prevalence was >60% in 3 consecutive monitoring events measured at least 4 weeks after the conclusion of releases, in order to reduce monitoring costs. The Jurujuba pilot release area in zone 1 is indicated with hatched shading. Maps were generated in ArcGIS 10.7 (Esri, Redlands, CA, USA) using base map and data from OpenStreetMap under open database license (CC BY-SA). (TIF) [file pntd.0009556.s005.tif]

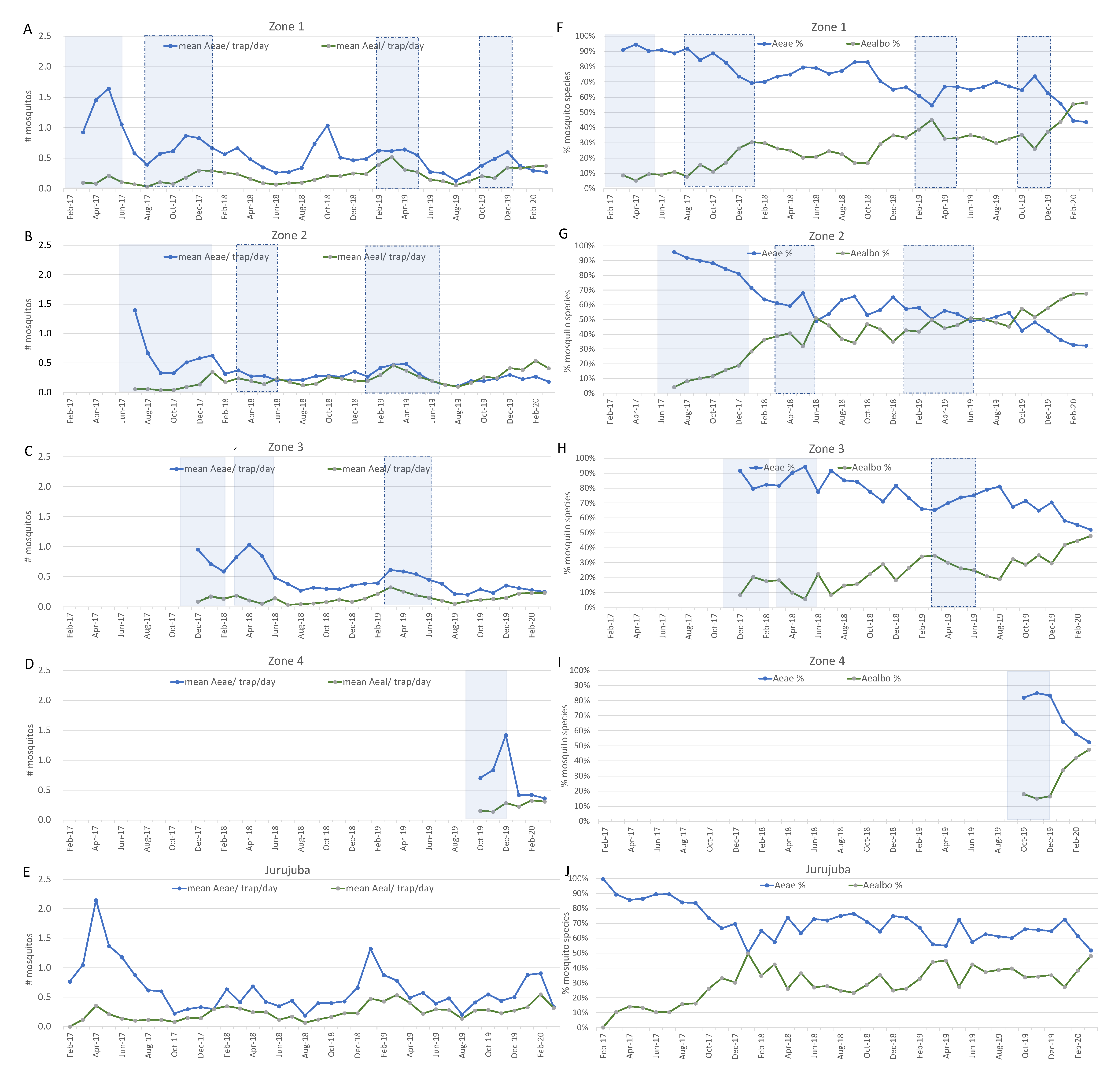

Supplement: S5 Fig — Panels A–E show the mean number of mosquitoes caught per trap per day for each species, each month, in release zones 1–4 and in the Jurujuba pilot release area. Panels F–J show the relative frequency of each species, each month, in release zones 1–4 and in the Jurujuba pilot release area. Shaded areas represent release periods. Dotted shaded areas indicate that only part of the zone was receiving releases. (TIFF) [file pntd.0009556.s006.tiff]

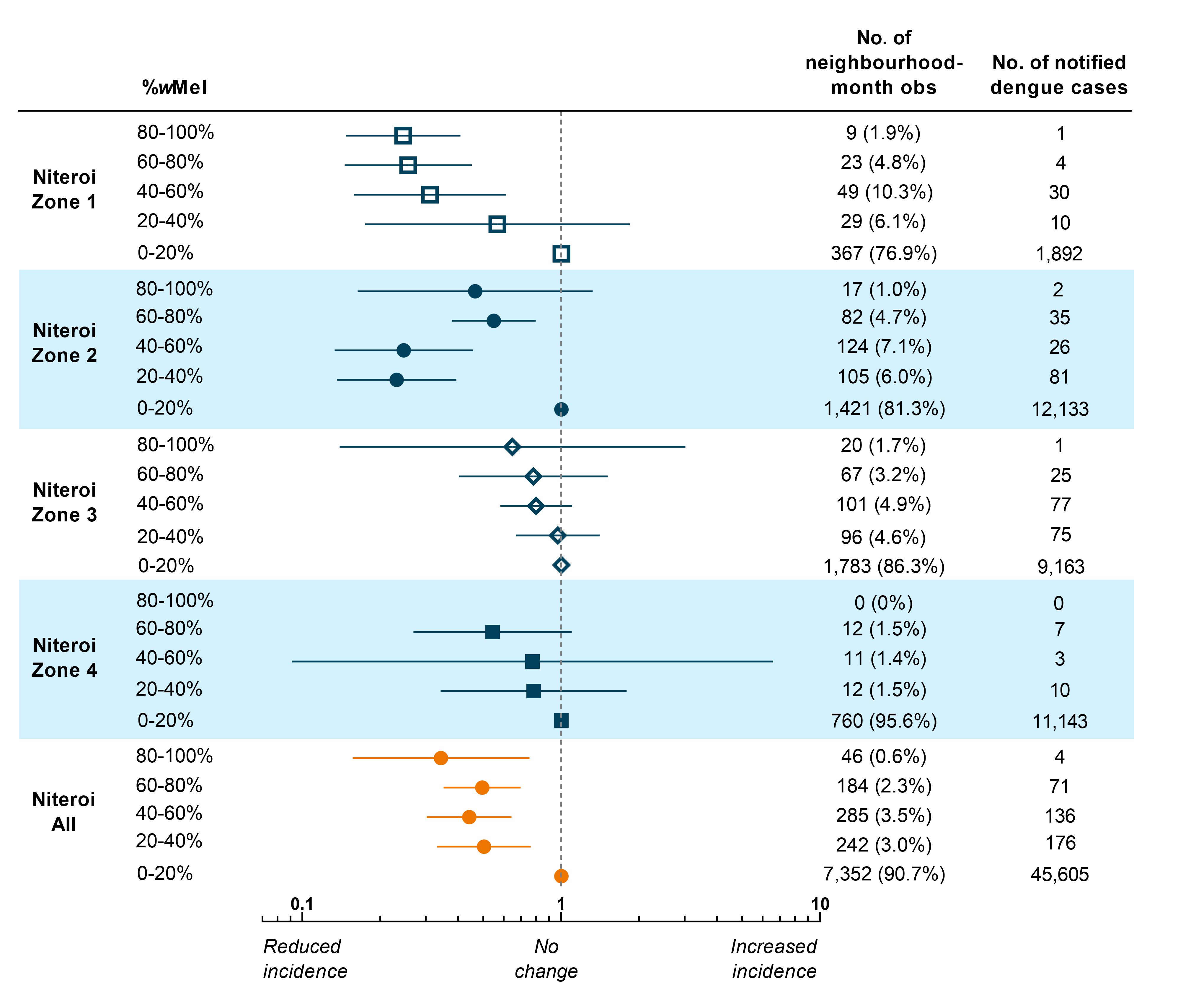

Supplement: S6 Fig — This analysis uses a three-month moving average of wMel% and excludes the Zone 1 pilot release area of Jurujuba. Point estimates (markers) and 95% confidence intervals (horizontal bars) are from controlled interrupted time series analysis of monthly dengue case notifications to the Brazilian national disease surveillance system (Jan 2007 –March 2020), by neighbourhood, in each release zone and in the aggregate release area. wMel prevalence was calculated as the percentage of trapped Ae. aegypti positive for wMel, in each neighbourhood each month, grouped by quintile. The lowest quintile (wMel 0–20%) served as the reference category for calculation of the incidence rate ratio (IRR) and included the monthly observations within that quintile from the respective release zone, as well as all observations from the untreated control zone (n = 3,021 neighbourhood-months observed and n = 11,278 notified dengue cases). (JPG) [file pntd.0009556.s007.jpg]

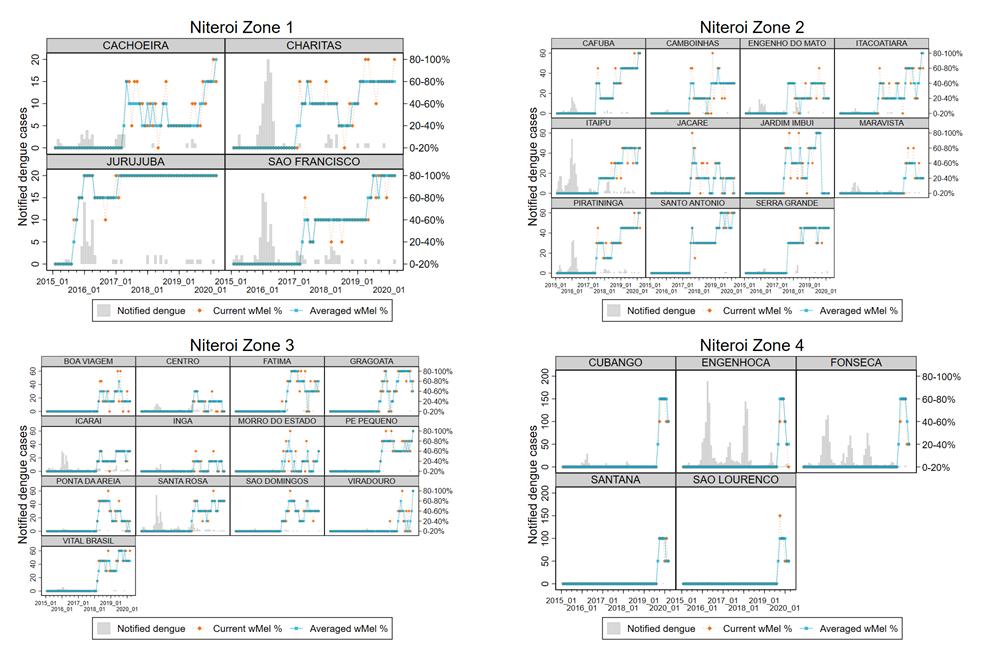

Supplement: S7 Fig — wMel% quintile was based on the wMel prevalence in a single month (current wMel%) or a three-month moving average (Averaged wMel%). (TIF) [file pntd.0009556.s008.tif]

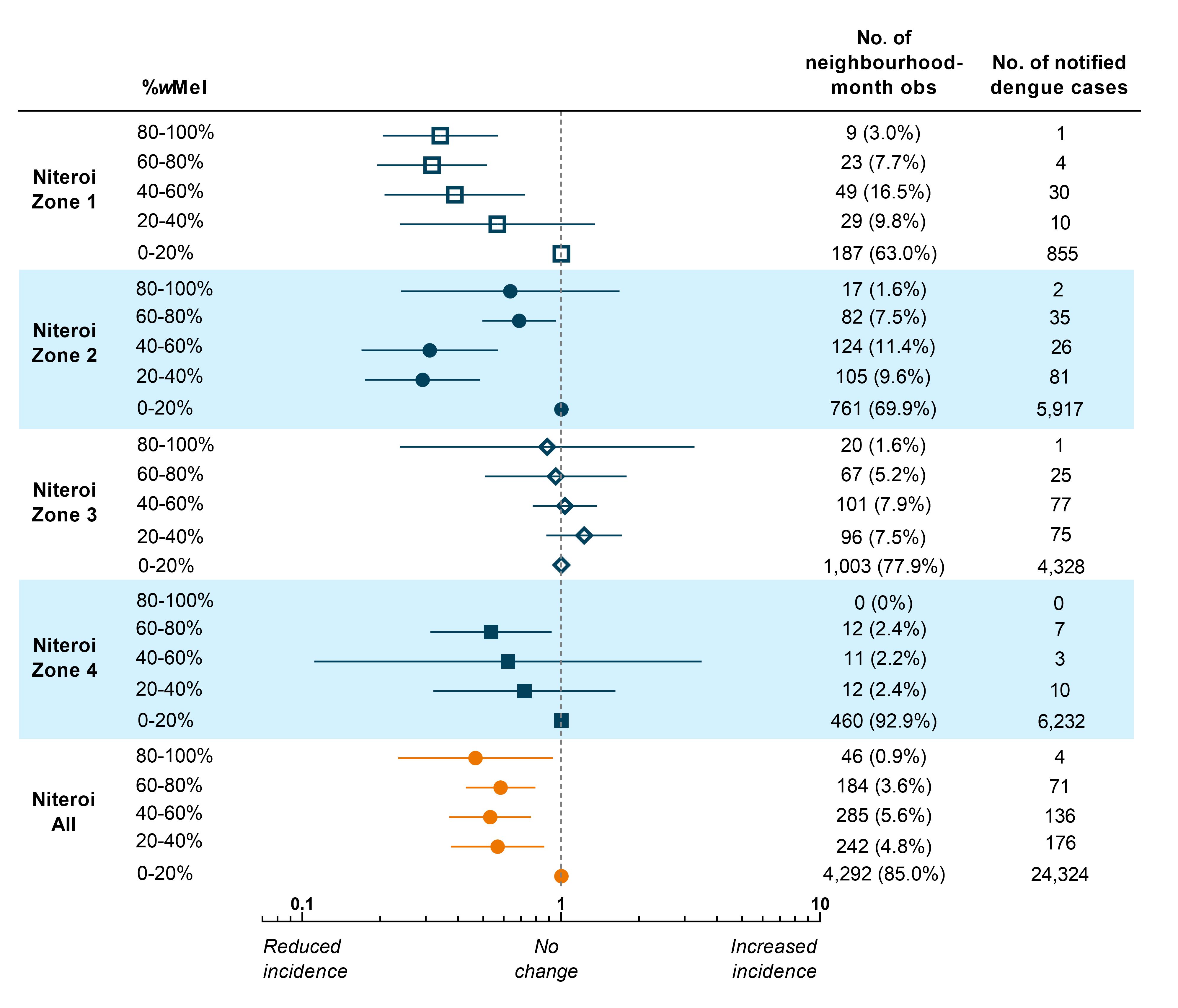

Supplement: S8 Fig — This sensitivity analysis excludes all observations prior to 2012, five years prior to the start of releases. Point estimates (markers) and 95% confidence intervals (horizontal bars) are from controlled interrupted time series analysis of monthly dengue case notifications to the Brazilian national disease surveillance system (Jan 2012 –March 2020), by neighbourhood, in each release zone and in the aggregate release area. wMel prevalence was calculated as the percentage of trapped Ae. aegypti positive for wMel, in each neighbourhood in a moving three-month window, grouped by quintile. The lowest quintile (wMel 0–20%) served as the reference category for calculation of the incidence rate ratio (IRR) and included the observations within that quintile from the respective release zone, as well as all observations from the untreated control zone (n = 1,881 neighbourhood-months observed and n = 6,996 notified dengue cases). (JPG) [file pntd.0009556.s009.jpg]
