# Supplementary material for: Effectiveness of Wolbachia-infected mosquito deployments in reducing the incidence of dengue and other Aedes-borne diseases in Niterói, Brazil: A quasi-experimental study
Source: PLoS Negl Trop Dis. 2021 Jul 12;15(7):e0009556. doi: 10.1371/journal.pntd.0009556 (PMC8297942; doi:10.1371/journal.pntd.0009556)
Supplement: S1 Table — IRRs are from negative binomial regression models of monthly case counts (Jan 2007 –June 2020 for dengue; Jan 2015 –June 2020 for chikungunya and Zika), with an offset for population size and 6-monthly flexible cubic splines to account for seasonal effects. The mixed effects model for the aggregate Niteroi release area included a random effect for release zone. (DOCX) [file pntd.0009556.s010.docx]

**S1 Table. Dengue, chikungunya and Zika incidence rate ratios in *Wolbachia*-release zones compared to the control zone.** IRRs are from negative binomial regression models of monthly case counts (Jan 2007 – June 2020 for dengue; Jan 2015 – June 2020 for chikungunya and Zika), with an offset for population size and 6-monthly flexible cubic splines to account for seasonal effects. The mixed effects model for the aggregate Niteroi release area included a random effect for release zone.

|  | Incidence rate ratio (95% confidence interval) | | |
| --- | --- | --- | --- |
|  | Dengue | Chikungunya | Zika |
| Zone 1 | 0.30 (0.19, 0.47) | 0.30 (0.15, 0.59) | 0.44 (0.18, 1.09) |
| Zone 2 | 0.24 (0.15, 0.38) | 0.93 (0.50, 1.73) | 0.66 (0.26, 1.63) |
| Zone 3 | 0.54 (0.37, 0.79) | 0.30 (0.17, 0.53) | 1.04 (0.50, 2.16) |
| Zone 4 | 0.31 (0.18, 0.54) | 0.29 (0.13, 0.67) | 0.60 (0.17, 2.05) |
| Niteroi | 0.31 (0.21, 0.46) | 0.44 (0.23, 0.84) | 0.63 (0.40, 0.99) |
