# Supplementary material for: Effectiveness of Wolbachia-infected mosquito deployments in reducing the incidence of dengue and other Aedes-borne diseases in Niterói, Brazil: A quasi-experimental study
Source: PLoS Negl Trop Dis. 2021 Jul 12;15(7):e0009556. doi: 10.1371/journal.pntd.0009556 (PMC8297942; doi:10.1371/journal.pntd.0009556)
Supplement: S2 Table — IRRs are from mixed effects negative binomial regression models of monthly dengue case counts (Jan 2007 –March 2020) by neighbourhood, with an offset for population size, 6-monthly flexible cubic splines to account for seasonal effects, and a random effect for neighbourhood. (DOCX) [file pntd.0009556.s011.docx]

**S2 Table. Dengue incidence rate ratios with increasing *w*Mel prevalence in *Aedes aegypti* populations in Niteroi neighbourhoods.** IRRs are from mixed effects negative binomial regression models of monthly dengue case counts (Jan 2007 – March 2020) by neighbourhood, with an offset for population size, 6-monthly flexible cubic splines to account for seasonal effects, and a random effect for neighbourhood.

|  | Incidence rate ratio (95% confidence interval) | | | | |
| --- | --- | --- | --- | --- | --- |
| *w*Mel% quintile | Zone 1 | Zone 2 | Zone 3 | Zone 4 | Niteroi |
| 0-20% | Ref | Ref | Ref | Ref | Ref |
| 20-40% | 0.56  (0.17, 1.83) | 0.23  (0.13, 0.39) | 0.96  (0.66, 1.40) | 0.78  (0.34, 1.78) | 0.50  (0.33, 0.76) |
| 40-60% | 0.31  (0.15, 0.61) | 0.24  (0.13, 0.45) | 0.79  (0.58, 1.09) | 0.77  (0.09, 6.57) | 0.44  (0.30, 0.64) |
| 60-80% | 0.25  (0.14, 0.45) | 0.54  (0.37, 0.79) | 0.77  (0.40, 1.51) | 0.54  (0.26, 1.09) | 0.49  (0.35, 0.69) |
| 80-100% | 0.24  (0.14, 0.40) | 0.46  (0.16, 1.32) | 0.64  (0.13, 3.01) | - | 0.34  (0.15, 0.75) |
