# Supplementary material for: Effectiveness of Wolbachia-infected mosquito deployments in reducing the incidence of dengue and other Aedes-borne diseases in Niterói, Brazil: A quasi-experimental study
Source: PLoS Negl Trop Dis. 2021 Jul 12;15(7):e0009556. doi: 10.1371/journal.pntd.0009556 (PMC8297942; doi:10.1371/journal.pntd.0009556)
Supplement: S3 Table — IRRs are from mixed effects negative binomial regression models of monthly dengue case counts (Jan 2012 –March 2020) by neighbourhood, with an offset for population size, 6-monthly flexible cubic splines to account for seasonal effects, and a random effect for neighbourhood. (DOCX) [file pntd.0009556.s012.docx]

**S3 Table. Dengue incidence rate ratios with increasing *w*Mel prevalence in *Aedes aegypti* populations in Niteroi neighbourhoods – sensitivity analysis** excluding pre-intervention observations prior to 2012 to achieve greater balance in the length of pre-intervention and post-intervention observation periods. IRRs are from mixed effects negative binomial regression models of monthly dengue case counts (Jan 2012 – March 2020) by neighbourhood, with an offset for population size, 6-monthly flexible cubic splines to account for seasonal effects, and a random effect for neighbourhood.

|  | Incidence rate ratio (95% confidence interval) | | | | |
| --- | --- | --- | --- | --- | --- |
| *w*Mel% quintile | Zone 1 | Zone 2 | Zone 3 | Zone 4 | Niteroi |
| 0-20% | Ref | Ref | Ref | Ref | Ref |
| 20-40% | 0.56  (0.23, 1.34) | 0.29  (0.17, 0.48) | 1.22  (0.87, 1.71) | 0.71  (0.32, 1.61) | 0.56  (0.37, 0.85) |
| 40-60% | 0.38  (0.20, 0.72) | 0.31  (0.16, 0.56) | 1.03  (0.77, 1.37) | 0.62  (0.11, 3.48) | 0.53  (0.36, 0.76) |
| 60-80% | 0.31  (0.19, 0.51) | 0.68  (0.49, 0.95) | 0.95  (0.50, 1.78) | 0.53  (0.31, 0.91) | 0.58  (0.42, 0.79) |
| 80-100% | 0.34  (0.20, 0.56) | 0.63  (0.24, 1.67) | 0.88  (0.23, 3.26) | - | 0.46  (0.23, 0.92) |
